# *Sentinel Amenable Mortality*: A New Way to Assess the Quality of Healthcare by Examining Causes of Premature Death for Which Highly Efficacious Medical Interventions Are Available

**DOI:** 10.1155/2018/5456074

**Published:** 2018-09-02

**Authors:** Montse Vergara-Duarte, Carme Borrell, Glòria Pérez, Juan Carlos Martín-Sánchez, Ramon Clèries, Maria Buxó, Èrica Martínez-Solanas, Yutaka Yasui, Carles Muntaner, Joan Benach

**Affiliations:** ^1^CAP El Clot and Unitat d'Avaluació, Sistemes d'Informació i Qualitat Assistencial, Gerència Territorial de Barcelona, Institut Català de la Salut, Departament de Salut, Generalitat de Catalunya, Barcelona, Spain; ^2^Agència de Salut Pública de Barcelona, Barcelona, Spain; ^3^CIBER Epidemiology and Public Health (CIBERESP), Barcelona, Spain; ^4^Universitat Pompeu Fabra, Barcelona, Spain; ^5^Sant Pau Biomedical Research Institute (IIB Sant Pau), Barcelona, Spain; ^6^Biostatistics Unit, Department of Basic Sciences, Universitat Internacional de Catalunya, Sant Cugat del Vallès, Spain; ^7^Pla Director d'Oncología, IDIBELL, Hospitalet de Llobregat, Barcelona, Spain; ^8^Departament de Ciències Clíniques, Universitat de Barcelona, Barcelona, Spain; ^9^Girona Biomedical Research Institute (IDIBGI), Salt, Spain; ^10^ISGlobal, Center for Research in Environmental Epidemiology (CREAL), Spain; ^11^Department of Epidemiology & Cancer Control (S6050), St. Jude Children's Research Hospital, Memphis, TN, USA; ^12^School of Public Health, University of Alberta, 4-274, Edmonton Clinic Health Academy, Edmonton, AB, Canada; ^13^Bloomberg Faculty of Nursing and Dalla Lana School of Public Health, University of Toronto, Canada; ^14^Health Inequalities Research Group-Employment Conditions Network (GREDS-EMCONET), Department of Political and Social Sciences, Universitat Pompeu Fabra, Barcelona, Spain; ^15^Johns Hopkins University, Public Policy Center, Barcelona, Spain; ^16^Grupo de Investigación Transdisciplinar sobre Transiciones Socioecológicas (GinTRANS2), Universidad Autónoma de Madrid, Madrid, Spain

## Abstract

**Background:**

Amenable mortality, or premature deaths that could be prevented with medical care, is a proven indicator for assessing healthcare quality when adapted to a country or region's specific healthcare context. This concept is currently used to evaluate the performance of national and international healthcare systems. However, the levels of efficacy and effectiveness determined using this indicator can vary greatly depending on the causes of death that are included. We introduce a new approach by identifying a subgroup of causes for which there are available treatments with a high level of efficacy. These causes should be considered sentinel events to help identify limitations in the effectiveness and quality of health provision.

**Methods:**

We conducted an extensive literature review using a list of amenable causes of death compiled by Spanish researchers. We complemented this approach by assessing the time trends of amenable mortality in two high-income countries that have a similar quality of healthcare but very different systems of provision, namely, Spain and the United States. This enabled us to identify different levels of efficacy of medical interventions (high, medium, and low). We consulted a group of medical experts and combined this information to help make the final classification of* sentinel amenable causes of death*.

**Results:**

* Sentinel amenable mortality* includes causes such as surgical conditions, thyroid diseases, and asthma. The remaining amenable causes of death either have a higher complexity in terms of the disease or need more effective medical interventions or preventative measures to guarantee early detection and adherence to treatment. These included cardiovascular diseases, diabetes, hypertension, all amenable cancers, and some infectious diseases such as pneumonia, influenza, and tuberculosis.

**Conclusions:**

* Sentinel amenable mortality* could act as a good sentinel indicator to identify major deficiencies in healthcare quality and provision and detect inequalities across populations.

## 1. Introduction

Healthcare systems, and the services they provide, represent a large economic expenditure for many countries and play an important role in public health. Healthcare accounts for around 10% of global GDP, which is mostly spent in high-income countries [1]. Research shows that a significant proportion of the global burden of disease could be reduced through effective medical treatment and prevention [2].

Steady advances in medical technology and knowledge must be subjected to a complex, continuous process of evaluation to ensure the efficacy and effectiveness of medical treatments and procedures [3, 4]. In 1976, Rutstein* et al*. introduced the concept of “sentinel health events” as a key indicator of the quality of healthcare services [5]. This indicator included a heterogeneous set of conditions (i.e., causes of unnecessary disease, preventable disability, and untimely death) that should not occur in an adequately functioning healthcare system. The most effective intervention (treatment, prevention, or both) was identified for each condition and, for some, restrictions affecting the avoidance of causes were also shown (e.g., specific circumstances, exposures, hazards, or age groups).

This seminal proposal was later revisited in the 1990s by Holland* et al*. [6], and more recently by Nolte* et al*. and Hoffmann* et al*. From this, the concept of “avoidable mortality” emerged as a new and useful measure to monitor the quality of healthcare services and assess the contribution of healthcare interventions to public health [7, 8]. Within this context, avoidable causes of death can be classified as either “preventable” or “amenable.” While preventable causes include conditions or causes for which premature death could be avoided through primary preventative interventions (e.g., national health promotion policies), amenable causes include conditions for which premature death could be prevented mainly through secondary or tertiary preventative interventions (e.g., medical care). This two-group classification scheme is particularly useful for researching and evaluating the impact of policies on population health. In fact, several studies have taken into account both groups of causes when analysing health inequalities associated with geographical patterns, social groups, or trends between and within countries [7–20].

All amenable causes have well-documented evidence supporting the efficacy of medical interventions in avoiding premature death. In this respect, efficacy is defined as “the ability of the science and technology of healthcare to bring about improvements in health when used under the most favourable circumstances.” [4] In the last decade, there have been numerous important medical and technological advances in the treatment and management of different causes of death. However, the degree of improvement in the efficacy of these medical interventions varies markedly between causes of death, ranging from high levels of efficacy for surgery on appendicitis and abdominal hernias, for example, to relatively low efficacy of interventions for ischemic heart disease and cerebrovascular diseases [7]. Thus, further analytical scrutiny of these causes needs to be performed to properly validate the “amenable mortality” concept [8, 21, 22]. For example, to date no study has systematically classified the causes of “amenable mortality” according to the level of efficacy of their respective available medical interventions.

Therefore, in this paper we introduce a new approach to amenable mortality by identifying “*sentinel amenable mortality”,* defined as amenable causes of death for which highly effective treatments are available. Such causes of death should be considered sentinel events that can help identify possible limitations in the effectiveness and quality of healthcare systems.

## 2. Methods

We used a list of 27 amenable causes of death that had been proposal by a group of Spanish experts [23] and later used in various empirical analyses [10, 24]. From this list, we excluded any adverse health events because they have a large variety of causes and are often studied separately as sentinel events themselves; this results in a final list of 26 amenable causes of death. In Table 1, we summarize the 26 causes of death, age groups, and ICD-9 and ICD-10 codes (International Classification of Disease; the Spanish list shows the correspondence between the two reviews for each cause of death).

We used two strategies to identify* sentinel amenable causes of death* based on the level of efficacy of the available medical interventions. First, we conducted an extensive literature review to compile updated medical and epidemiological knowledge on the efficacy of medical procedures to avoid amenable causes of death, considering “efficacy” as the capacity to produce a desired result or effect under the best conditions. Additionally, we complemented this strategy by assessing the time trends of these causes of death in two high-income countries, namely, Spain and the United States. Although the quality of healthcare offered in these two countries is very similar (i.e., both are based on advanced medical knowledge and technology), they have very different healthcare systems. By performing a comparative analysis of the mortality rates in Spain and the United States, we were able to assess the similarities and divergence in the distribution. Second, we assessed the efficacy of these interventions by consulting a group of medical experts. Our final intervention efficacy-based classification of amenable causes of death was obtained from a consensus of both strategies (i.e., high or medium-high efficacy in Strategy 1; and In Strategy 2, >85% of experts assign the cause to the high efficacy group).

### 2.1. Strategy 1: Classification of Amenable Causes of Death through Literature Review and Empirical Analysis

For each of the 26 selected amenable causes of death, we gathered the most up-to-date medical evidence and used it to determine the degree of efficacy of the available interventions in avoiding premature mortality. Specifically, we conducted a review of clinical and epidemiological scientific literature, examining various sources of health information including articles, books, reports, and websites. We surveyed publications from the years 2000 to 2016 by using search terms such as “efficacy”, “intervention”, “health care”, “treatment”, “poor outcomes”, and “mortality”, and by citing the specific cause of death. This first phase of the literature review yielded 390 relevant publications. We then filtered these publications by applying a set of exclusion criteria. First, we excluded any publication that was not eligible according to information in the title or abstract (n = 212), or full-text (n = 88). Second, we excluded any publication that did not contain relevant information for classifying the causes of death based on intervention efficacy (n = 43). In the end, we selected a total of 47 information sources to help identify which of the 26 causes of death are* sentinel amenable causes*.

**Figure 1:**
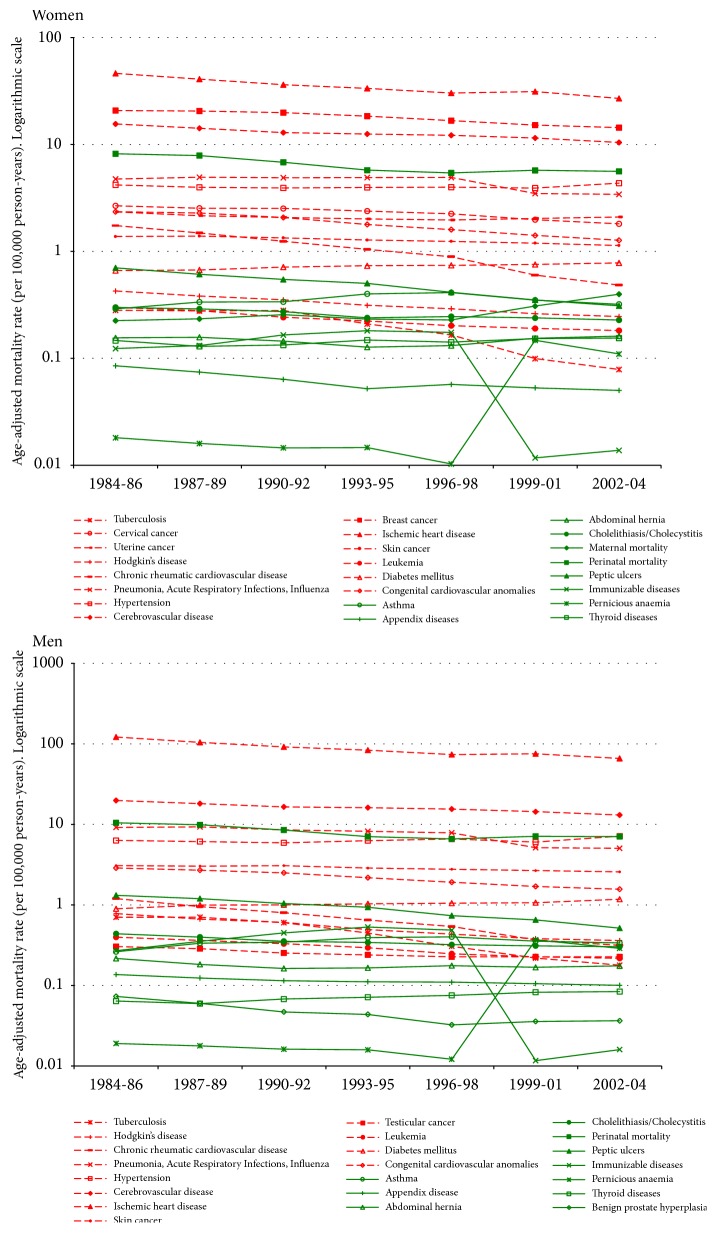
Evolution of amenable causes of death by sex, United States, 1984-2004. Colours in the figure: medium/low efficacy group (red) and high efficacy group (green).

**Figure 2:**
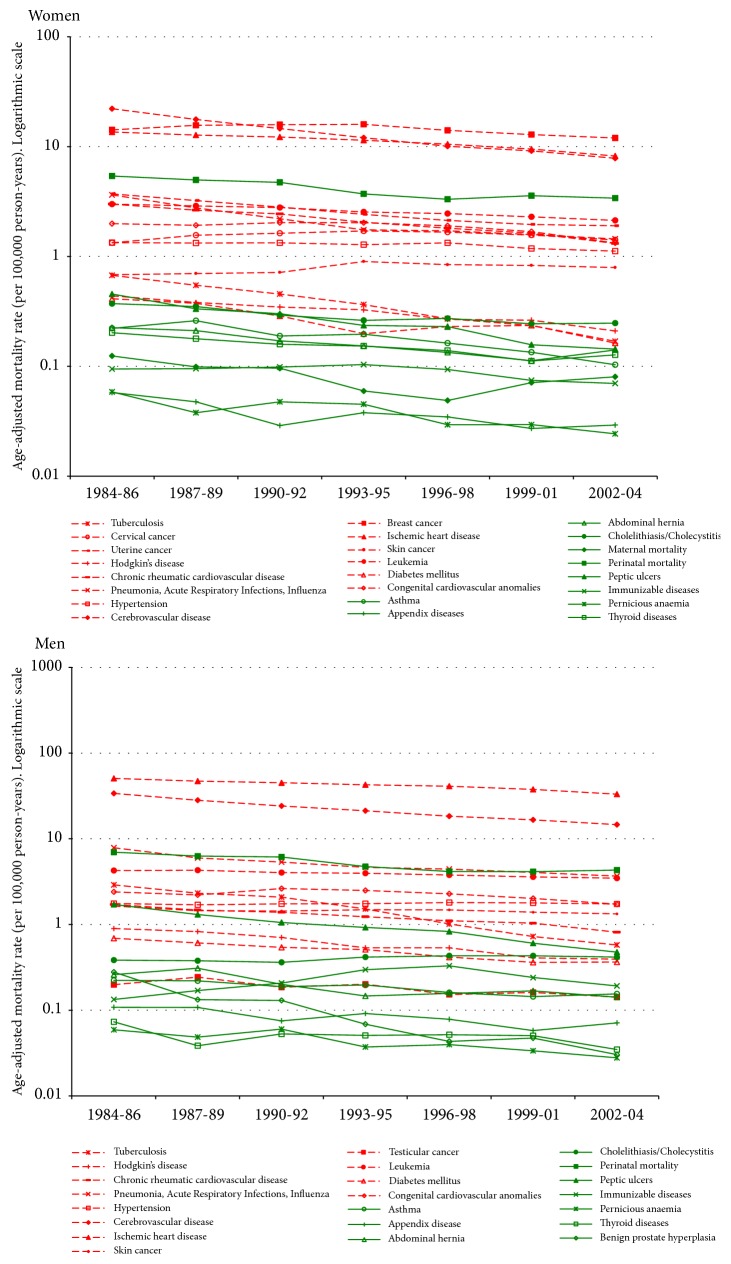
Evolution of amenable causes of death by sex, Spain, 1984-2004. Colours in the figure: medium/low efficacy group (red) and high efficacy group (green).

Based on this literature review, we classified causes of death as having (i) a high level of intervention efficacy (G1); (ii) a low level of intervention efficacy (G2); or (iii) an uncertain degree of intervention efficacy, with priority given to high efficacy (G1/G2) or with priority given to low efficacy (G2/G1). Next, we analysed and compared changes in mortality rates for these three different groups of amenable causes of death. Using the World Health Organization (WHO) standard population [25], we calculated direct standardized mortality rates per 100,000 individuals in Spain and the United States for 3-year periods spanning 1984 to 2004. We obtained the mortality and population data for each year, age group, and sex from the Spanish National Institute of Statistics (INE) [26] for the Spanish analysis and from the Centers for Disease Control (CDC) [27] and the US Census Bureau [28] for the United States analysis. Changes in mortality rates for each of the 26 causes of death and mortality rates for the last period (2000-2004) are shown, respectively, in Figures 1 and 2 and in Table 2.

### 2.2. Strategy 2: Classification of Amenable Causes of Death according to the Opinions of Medical Experts

We conducted in-depth interviews with 11 medical practitioners from a university/tertiary hospital in Barcelona, Spain. These were originally conducted in 2009 but also revisited in 2014. We conducted the interviews (15 to 30 minutes) in the hospital. The medical experts were mid-career doctors, 4 women and 7 men, specialized in internal medicine (n = 4), infectious diseases (n = 2), surgery and trauma (n = 2), anaesthesiology (n = 1), paediatrics (n = 1), and quality of healthcare services (n = 1). After asking for an overall assessment of the 26 amenable causes of death and the efficacy levels of their associated medical interventions, we asked the following specific question: “Which amenable causes of death in the list could be avoided in a high percentage of cases given currently available knowledge and technology used in medical interventions in wealthy countries?” Using the answers to the above question, we calculated the percentage of experts that classified each amenable cause of death as belonging to the group with the highest level of intervention efficacy.

### 2.3. Combined Consensus Using Both Strategies

Finally, according to the efficacy of their respective medical interventions, we classified the causes of death as either high efficacy or medium-low efficacy. In the high efficacy group, we included only those causes of death that had been classified as having a high intervention efficacy in both strategies.

## 3. Results

Table 3 summarizes the results obtained for the classification of deaths amenable to medical intervention using the two distinct strategies.

### 3.1. Strategy 1: Classification of Amenable Causes of Death through Literature Review and Empirical Analysis

#### 3.1.1. Literature Review

For all amenable causes of death in the list, we found evidence concerning the efficacy of their respective medical interventions [29, 30, 39]. In the assessment of intervention efficacy, age groups are of special concern [7, 9, 12]. While for most causes of death the amenability of mortality is restricted to individuals aged 0-74 [7, 9, 12, 23], different age classifications have been discussed for some conditions (described below).

After reviewing all available medical, scientific, and technological information, we classified amenable causes of death into two groups.

The* first group, *labelled as G1 (“amenable causes with medical interventions of* high efficacy*”), includes those causes for which there is strong evidence of highly efficacious medical interventions. This is clearly the case in many surgical conditions such as appendicitis, abdominal hernias, peptic ulcers, cholelithiasis/cholecystitis, and benign prostate hyperplasia. Taking into account advances in surgical techniques (e.g., laparoscopy and other noninvasive forms of surgery), pharmacotherapy, and medical management of complications, mortality resulting from these conditions should be extremely low in most age groups. Thus, amenability should only be age-restricted in cases when there are also nonprevalent medical complications [9, 29, 30]. There is also not much doubt regarding the high efficacy of medical management for the most prevalent thyroid disorders and asthma [12, 29, 30, 39, 40]. However, some authors recommend restricting the amenability of asthma-related mortality to middle age groups because of difficulties in distinguishing its diagnosis as a cause of death among middle-aged adults and the elderly [12, 23]. There is also a strong consensus to not consider mortality due to infectious diseases such as tetanus, diphtheria, whooping cough, poliomyelitis, measles, and rubella, since there are effective vaccines for all of these. Another condition that should be highly amenable is pernicious anaemia (i.e., anaemia due to deficiencies in iron, B12 vitamins, proteins, or other nutritional anaemia), unless there is also a severe underlying disease such as cancer [29, 30]. Maternal mortality, which includes numerous causes of death, should also be largely avoidable if the mother is provided with adequate obstetric and prenatal care [31–33]. In fact, mortality due to this condition is considered rare in high-income countries and, together with perinatal mortality, is one of the most relevant indicators of health and quality in healthcare systems. The efficacy of medical interventions in the management of perinatal deaths is, however, more controversial [34–36]. While the two conditions involve a complex set of medical interventions that aim to avoid death, considering the overall levels of efficacy achieved in high-income countries for conditions related to both maternal and perinatal mortality should be included in the high efficacy group.

The* second group *contains the remaining amenable causes of death which, according to current knowledge and technology, have lower levels of intervention efficacy. We call this group G2: “amenable causes with medical interventions* of medium-low efficacy*”. In this group, we included causes of death such as cardiovascular diseases (ischemic heart disease, IHD) and cerebrovascular diseases, which are highly prevalent in the population and are generally considered to be only partly amenable to current medical interventions (i.e., pharmacotherapy, lifestyle modifications and adherence to treatment) [7]. Nonetheless, the efficacy of the treatment and management of these causes have improved considerably in recent years [7, 9, 29, 30, 67]. These diseases require urgent treatment and highly specialized health care, such that having good accessibility to IHD and stroke units is highly important. They should also be tackled from a public health perspective (i.e., primary prevention) to help reduce risk factors. We also included diabetes, pneumonia, and hypertension in this group [9, 29, 30, 53, 56–59]. While mortality due to diabetes is considered amenable until middle age (about 50 years), patients with type 1 diabetes can also suffer major complications at a younger age [53]. Hypertension, pneumonia, acute respiratory infections, and influenza are conditions with a high level of nonamenable mortality in the presence of comorbidity [9, 29, 30, 56, 57], especially in the elderly or in people with depressed immune systems.

All cancers in the list of amenable causes were also included in this second group. Although patients with Hodgkin's disease and testicular cancer now have increased survival rates (>5 years) [62, 64], there are many factors such as the nature of the disease, early detection, stage of the cancer, toxicity of treatments, and metastatic processes that can critically influence their outcome and complete cure rate [60–64, 66, 68]. These factors also affect other cancers such as breast and cervical cancer despite significantly increased knowledge regarding their etiology, treatment, and early detection through screening programs [61, 68]. Prognosis and medical achievements are even less favourable in relation to other forms of cancer, such as uterine or skin cancer [29, 30, 62].

Based on our literature review, we were unsure how to best classify conditions such as cardiovascular congenital anomalies and chronic rheumatic heart disease. Although there have been many advances in surgical and medical interventions aimed at avoiding premature deaths due to these conditions, the prognosis or the degree of efficacy of the interventions depends on the type of anomaly [9, 29, 30, 51, 52]. In the case of chronic rheumatic heart disease, we must consider a change in its etiology. While improvements in pharmacotherapy and antibiotics reduce rheumatic fever-related cases, other forms of heart-valve disease are emerging as a consequence of increased life expectancy [51]. Thus, premature deaths caused by this disease should only be considered highly amenable to currently available interventions when dealing with younger age groups.

Finally, we also included tuberculosis in this second group, even though its etiology, incidence, and prevalence make its classification more controversial. Tuberculosis is an old infectious disease that still constitutes an important public health problem worldwide [44, 45]. After the identification of this bacillus and the subsequent development of appropriate pharmacotherapy, this condition was considered highly treatable by health services. In recent decades, however, some well-known factors, such as having a depressed immune system and, in particular, the presence of AIDS, have been found to result in a poor prognosis [46]. Moreover, in the last few years, additional complications have emerged, including the spread of multidrug-resistant strains mainly due to poor patient compliance with treatment [47–50]. These emerging problems diminish the level of efficacy of current medical treatments and urgently demand further advances in the knowledge and management of this disease.

#### 3.1.2. Empirical Analysis

**Figure 3:**
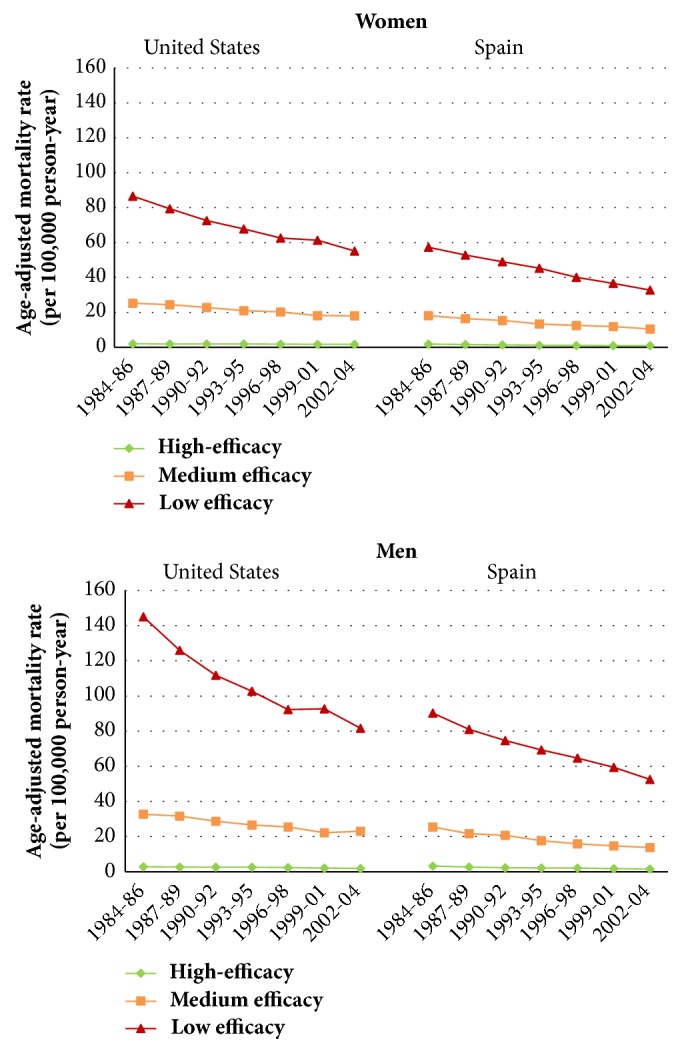
Evolution of age-adjusted amenable mortality rate by efficacy group and sex. United States and Spain, 1984-2004. High efficacy: amenable causes with medical interventions of* high efficacy *(G1); low efficacy: amenable causes with medical interventions of* low efficacy (*G2); and medium efficacy: uncertain level of efficacy but priority given to the first (G1/G2 or G2/G1). Three-way categories were based exclusively on the literature review.

In Figure 3, we show the evolution of age-adjusted mortality rates from 1984 to 2004 for three groups of amenable causes of death, for both women and men in Spain and the United States (see Figures 1 and 2 for more detailed information about the time trends of each of the 26 amenable causes of death). Classification of the causes of death into the three groups was based on the efficacy levels of their respective treatments as determined by the literature review (Table 3, Strategy 1): high (G1 or G1/G2), medium efficacy (G2/G1), and low efficacy (G2)).

While the time trend of mortality rates is very similar in both countries, we observe higher mortality rates in the United States than in Spain for the low efficacy group in both sexes and for the medium efficacy group in men. In particular, we also observe that the most pronounced decline in age-adjusted mortality rates is for the low efficacy group. On the other hand, those amenable causes of death belonging to the high efficacy group present the lowest rates of mortality.

### 3.2. Strategy 2: Classification of Amenable Causes of Death according to the Opinions of Medical Experts

A very high percentage of the medical experts (more than 85%) classified maternal and perinatal causes of mortality, surgical conditions, pernicious anaemia, and thyroid and immunizable diseases into the* high efficacy *group. In contrast, only a medium (50-85%) or low (<50%) percentage of experts classified cardiovascular diseases (including congenital cardiovascular anomalies) and most cancers into the* high efficacy *group. Finally, there was some disagreement regarding the classification of chronic rheumatic heart disease, pneumonia, hypertension, and diabetes, as only a medium level of agreement was reached.

### 3.3. Combined Consensus Using Both Strategies

For most causes of death, we obtained similar results between strategies. Those amenable causes of death for which only weak evidence was found to classify them into the* high efficacy *group were classified into the* medium-low efficacy *group (hypertension, diabetes mellitus, chronic rheumatic heart disease, and congenital cardiovascular diseases).

Based on the literature review alone, it was not clear how to classify perinatal mortality. Consequently, we decided to follow the criteria of the medical experts. A similar situation occurred for certain cancers (cervical cancer, testicular cancer, and Hodgkin's disease). In these cases, however, given the low degree of agreement reached by the experts, we placed them into the* medium-low efficacy* group. Finally, we also decided to keep tuberculosis in the* medium-low efficacy* group because of the aforementioned limitations uncovered in the literature review.

## 4. Discussion

In this study, we propose a new way to classify causes of avoidable premature mortality which are amenable to medical care. Using available scientific and medical knowledge, we show that it is possible to differentiate between causes of death based on the level of efficacy of their respective medical interventions. To our knowledge, this is the first proposal that aims to classifying amenable causes of death in accordance with the efficacy of medical interventions.

Using this approach, we have obtained a highly specific and homogeneous group of amenable causes of death for which medical interventions are highly efficacious. These causes of death, hereafter* sentinel amenable mortality, *are useful for assessing the quality of healthcare services as a* sentinel indicator*.

For the final classification, we decided to classify causes of death into one of two categories:* high *or* medium-low level of efficacy*. Importantly, this selection was based on conservative criteria, so that if we were unsure about the level of efficacy of a medical intervention, the respective cause of death was classified into the* medium-low* category.

For the purpose of making this two-group classification scheme, we combined several different information-seeking strategies (literature review, empirical analysis, and expert assessment) instead of performing just a systematic review. There were two main reasons behind this multistrategy approach. First, it is difficult to categorize evidence on the efficacy of medical interventions since the available evidence is both voluminous and highly variable for each cause of death. Second, we wanted to reach a deeper understanding by identifying information that is important for distinguishing between the efficacy levels of medical interventions.

Empirical analysis, showing time trends in age-adjusted mortality rates in Spain and the United States, was useful not only for assessing the similarities and divergence in the distribution of each amenable cause of death, but also for performing the preliminary categorization into the three different efficacy groups. Despite large differences between the national health systems of Spain and the United States, the two countries are similar in terms of* efficacy* (i.e., healthcare performance), in that they both employ advanced medical knowledge and technologies; however, they are quite different in the* effectiveness* of the interventions, for example, in terms of accessibility.

The overall consistency in the distribution and evolution of mortality rates in both Spain and the United States reinforces our hypothesis that the causes of amenable mortality are heterogeneous. This in turn justifies our use of intervention efficacy as a mode of classification. Indeed, the amenable causes of death that we classified into the highest efficacy group generally showed the lowest mortality rates in both countries (data shown in Figures 1 and 2), albeit with a few exceptions.

In the* high efficacy *group, perinatal mortality in both sexes and peptic ulcers in men showed higher mortality rates than the other amenable causes in this group. One possible reason for the divergence in perinatal mortality could be the presence of biases in the mortality records. Some studies have shown that international differences in published perinatal mortality rates may partly reflect differences in the criteria used to record and publish them [37, 38]. However, since we observed similar results in both countries, the high mortality due to this condition is not likely to be a categorization issue. In fact, we found that a high percentage of medical experts agreed on classifying perinatal mortality into the highest efficacy group. Thus, despite the availability of highly efficacious interventions for perinatal mortality, the high mortality rates in high-income countries could be explained by a lack of effectiveness of healthcare services [34]. Moreover, in high-income countries, high mortality rates are more strongly associated with a lack of access to healthcare than with the degree of efficacy of the medical interventions themselves [35, 36]. With respect to peptic ulcers, we also observed high mortality rates during the period studied. In this case, we should not only consider potential deficiencies in the effectiveness of medical interventions, but also the presence of severe complications that could limit the amenability of this cause of death [41–43].

Within the* medium-low efficacy *group, we observed a generalized decline of amenable mortality, probably due to achievements in the effectiveness of medical interventions but also to social determinants of health. We found exceptions in both countries. First, in the case of Hodgkin's disease and testicular cancer, mortality rates were very low in comparison to the rest of the amenable causes. Clearly, noticeable achievements in the management of these cancers have been made in recent years, and ultimately these have led to a considerable increase in the cure rate among patients. Therefore, these two cancers could actually be considered to belong to the highest efficacy group. In our list, however, we decided not to include these causes in the* high efficacy *group because their treatments have limited efficacy at later stages of the disease and these diseases are often characterized by metastasis [65]. Additionally, studies showing better results in the prognosis of testicular cancer and Hodgkin's disease are mainly based on survival indicators. In particular, it is worth noting that Hodgkin's disease survival is only better in the early stages [60, 64]. Second, we also observed considerably low mortality for diabetes in both countries, especially in Spain. For this condition, we only selected cases in which the patient was aged under 50. Although the efficacy of medical interventions applied to this age group might be higher, type 1 diabetes may still present complications at earlier ages. On the other hand, we must consider limitations in the registration of mortality data for this condition [54, 55], which may partly explain lower mortality rates. In the end, we decided to assign this condition to the lower efficacy group because it is an important risk factor for major complications, especially in the presence of comorbidity. Finally, for tuberculosis, we also observed lower mortality rates and specifically a significant decrease in the rates over the study period for both countries, particularly among women. For this condition, high levels of effectiveness have been achieved in high-income countries. Nevertheless, the burden of other related diseases such as AIDS, the extensive emergence of multidrug-resistant strains, and the difficulty in enforcing treatment compliance make it necessary to develop new treatments to ensure both greater efficacy and effectiveness in the presence of medical complications. [46–50]

We mention some potential limitations of our study. We used a standardized list of amenable causes of death created by Spanish researchers. Although this list is not very different compared to other lists found in the literature, it does exclude certain causes of death, such as colon cancer, nephritis and nephrosis, and epilepsy that appear in other international lists [7, 8] and in particular, in recent studies conducted in the United States [69]. Nonetheless, the fact that there is a discussion about whether these causes of death should be included in the list is another reason to categorize them into the lowest efficacy group.

For most amenable causes of death, we considered the standard age group to be those patients aged between 0 and 74 years. In recent years, improvements in disease management have been introduced both for the youngest groups and among the elderly. However, taking into account the burden of comorbidity and difficulties in managing some diseases among the elderly, we should discuss whether the efficacy of some medical interventions should only be considered high for those under 65. This is the case, for instance, in chronic rheumatic heart disease and the emerging forms of less-amenable valve diseases at older ages [52]. For a few causes of death such as peptic ulcers and tuberculosis, we observed some differences between the sexes. These differences might be due to the etiology of the diseases or may even be related to limitations in* efficacy* [29, 30]. Thus, in the future it may be worthwhile to classify amenable causes of death according to sex and not only in relation to the efficacy of medical interventions [70].

With respect to the empirical analysis, it is important to consider the quality and variability of the certification and coding of the underlying causes of death, or in other words, changes to the classification of ICD codes and interpretation of the data. With regard to the former issue, the overall quality of mortality data has been recognized as reasonable for both countries [71, 72]. In terms of variability, we should consider that differences in mortality rates and trends could be due to the varying* effectiveness *of medical interventions and to socioeconomic or environmental determinants. For rare amenable causes of death, case-fatality rates, rather than population-based mortality rates, might help to clarify the efficacy of relevant interventions. Nevertheless, incidence data are not easily accessible, and analysing a specific study for each amenable cause of death falls far from the scope of this study. It could, however, be the next step in future studies examining the quality of healthcare after an increment in the* sentinel amenable mortality* indicator is detected.

Finally, consulting a group of medical experts to help classify causes of death has the intrinsic limitation of bias in selecting its members. However, the group we chose came from a public university hospital in Spain and was composed of well-informed medical experts who were specialized in interventions related to the considered causes of death.

In this study, we show that it is possible to separate out those causes of death that are highly amenable in all countries. As we mentioned above, such causes could be considered* sentinel health events *and could help in assessing the quality of health services worldwide. As we found lower mortality rates in both countries for those amenable causes of death in the* high efficacy *group, high mortality rates or any such events due to these causes are more clearly related to the effectiveness of health services.

Amenable causes of death that were classified into the* medium-low efficacy *group are still a great challenge for science, technology, and medicine. With respect to these causes, the development of more effective public healthcare systems and interventions that take into account environmental, social, and economic factors will prove critical in the future.

Divergence between the theoretical and empirical results obtained in our study may suggest some limitations with respect to our proposed classification method and/or issues related to the effectiveness of healthcare services. To our knowledge, this is the first ever proposal to classify amenable causes of death according to the efficacy level of their respective interventions. To validate our proposal, similar initiatives in different contexts or using different approaches are needed. We should also consider, however, the potential inequalities or difficulties of healthcare services in terms of patients being able to access highly efficacious medical interventions. Besides being affected by the characteristics of public health and healthcare systems, this accessibility issue is also influenced by other medical, environmental, political, and economic factors.

In conclusion, the separation of* sentinel events of amenable causes of death*, or those whose medical interventions have* high efficacy*, might be useful for measuring differences and anomalies in healthcare performance. This distinction could therefore be a good indicator for detecting inequalities among populations served by different health services both within and between countries. This novel way of classifying* amenable mortality *can also help to accurately pinpoint the degree to which different causes of death can be avoided. Furthermore, it is also useful in improving the knowledge required to conduct studies on the effectiveness of healthcare services, studies which ultimately aim to reduce mortality induced by these causes. Finally, this method should be continuously discussed and updated in accordance with new medical and technological advances.

## Figures and Tables

**Table 1:** Avoidable premature causes of death amenable to medical intervention [21].

Cause of death	Age	ICD-9	ICD-10
Tuberculosis	0-74	010-018 137	A15-A19, B90

Immunizable diseases	0-74	032, 033, 037, 045, 055, 056, 070.0, 070,1, 070.2-070.3, 072	A35, A36, A37, A49.2, A80, B05, B06, B15, B16, B17,0, B18,0-B18,1, B26

Pneumonia, acute respiratory infections, influenza	0-74	460-466, 480-486, 487	A48.1, J00-J06 (except J02.0, J03.0), J10-J11, J12-J18 (except J18.2), J20-J22

Skin cancer (melanoma and no-melanoma)	0-74	172,173	C43, C44, C46,0, C46,9

Breast cancer (women)	0-74	174	C50

Cervical cancer	15-74	180	C53

Uterine cancer	15-74	182, 179	C54, C55

Testicular cancer	0-74	186	C62

Hodgkin's disease	0-74	201	C81

Leukemia	0-14	204-208	C91-C95

Pernicious anemia	0-74	280-281	D50-D53

Thyroid diseases	0-74	240-246	E00-E07

Diabetes mellitus	0-49	250	E10-E14

Cerebrovascular diseases	0-74	430-438	G45, F01.1, I60-69,

Chronic rheumatic cardiovascular disease	0-74	393-398	I05-I09

Hypertension	0-74	401-405	I10-I15

Ischemic heart disease	35-74	410-414	I20-I25

Asthma	5-49	493	J45-J46

Peptic ulcers	0-74	531-534	K25-K28

Appendix diseases	0-74	540-543	K35-K38

Abdominal hernia	0-74	550-553	K40-K46

Cholelithiasis/cholecystitis	0-74	574-575	K80-K82

Benign prostate hyperplasia	0-74	600	N40

Perinatal mortality		760-779	P00-P96, A33

Maternal mortality	All	630-676	O00-O99, A34

Congenital cardiovascular anomalies	0-74	745-747	Q20-Q28, I51.0

Adverse events occurred during medical and surgical intervention	All	E870-879	Y60-Y84

ICD: international classification of diseases; data sorted by ICD-10th codes.

**Table:** (a) Women

**Cause of death**	**Age group **	**ICD-9**	**ICD-10**	**United States**	**Spain**
Ischemic heart disease	35-74	410-414	I20-I25	26,96	8,20

Breast cancer	0-74	174	C50	14,36	11,97

Cerebrovascular diseases	0-74	430-438	G45, F01.1, I60-69,	10,45	7,82

Perinatal mortality		760-779	P00-P96, A33	5,61	3,40

Hypertension	0-74	401-405	I10-I15	4,34	1,12

Pneumonia, acute respiratory infections, influenza	0-74	460-466, 480-486, 487	A48.1, J00-J06 (except J02.0, J03.0), J10-J11, J12-J18 (except J18.2), J20-J22	3,42	1,45

Uterine cancer	15-74	182, 179	C54, C55	2,10	1,90

Cervical cancer	15-74	180	C53	1,81	1,41

Congenital cardiovascular anomalies	0-74	745-747	Q20-Q28, I51.0	1,27	1,33

Skin cancer (melanoma and no-melanoma)	0-74	172,173	C43, C44, C46,0, C46,9	1,13	0,79

Diabetes mellitus	0-49	250	E10-E14	0,78	0,16

Chronic rheumatic cardiovascular disease	0-74	393-398	I05-I09	0,48	1,31

Maternal mortality	All	630-676	O00-O99, A34	0,40	0,08

Asthma	5-49	493	J45-J46	0,32	0,10

Peptic ulcers	0-74	531-534	K25-K28	0,31	0,14

Hodgkin's disease	0-74	201	C81	0,25	0,21

Cholelithiasis/cholecystitis	0-74	574-575	K80-K82	0,23	0,25

Leukemia	0-14	204-208	C91-C95	0,18	2,13

Abdominal hernia	0-74	550-553	K40-K46	0,16	0,14

Thyroid diseases	0-74	240-246	E00-E07	0,15	0,13

Pernicious anemia	0-74	280-281	D50-D53	0,11	0,02

Tuberculosis	0-74	010-018 137	A15-A19, B90	0,08	0,17

Appendix diseases	0-74	540-543	K35-K38	0,05	0,03

Immunizable diseases	0-74	032, 033, 037, 045, 055, 056, 070.0, 070,1, 070.2-070.3, 072	A35, A36, A37, A49.2, A80, B05, B06, B15, B16, B17,0, B18,0-B18,1, B26	0,01	0,07

Data sorted descendent by age-standardized mortality rates in the United States.

**Table:** (b) Men

**Cause of death**	**Age group**	**ICD-9**	**ICD-10**	**United States**	**Spain**
Ischemic heart disease	35-74	410-414	I20-I25	65,79	33,12

Cerebrovascular diseases	0-74	430-438	G45, F01.1, I60-69,	13,08	14,64

Hypertension	0-74	401-405	I10-I15	7,16	1,73

Perinatal mortality		760-779	P00-P96, A33	7,06	4,32

Pneumonia, acute respiratory infections, influenza	0-74	460-466, 480-486, 487	A48.1, J00-J06 (except J02.0, J03.0), J10-J11, J12-J18 (except J18.2), J20-J22	5,05	3,67

Skin cancer (melanoma and no-melanoma)	0-74	172,173	C43, C44, C46,0, C46,9	2,57	1,33

Congenital cardiovascular anomalies	0-74	745-747	Q20-Q28, I51.0	1,57	1,73

Diabetes mellitus	0-49	250	E10-E14	1,18	0,37

Peptic ulcers	0-74	531-534	K25-K28	0,52	0,48

Hodgkin's disease	0-74	201	C81	0,36	0,39

Asthma	5-49	493	J45-J46	0,34	0,16

Chronic rheumatic cardiovascular disease	0-74	393-398	I05-I09	0,32	0,81

Cholelithiasis/cholecystitis	0-74	574-575	K80-K82	0,30	0,42

Pernicious anaemia	0-74	280-281	D50-D53	0,29	0,03

Testicular cancer	0-74	186	C62	0,23	0,14

Leukemia	0-14	204-208	C91-C95	0,22	3,48

Tuberculosis	0-74	010-018 137	A15-A19, B90	0,18	0,58

Abdominal hernia	0-74	550-553	K40-K46	0,17	0,14

Appendix diseases	0-74	540-543	K35-K38	0,10	0,07

Thyroid diseases	0-74	240-246	E00-E07	0,08	0,03

Benign prostatic hyperplasia	0-74	600	N40	0,04	0,03

Immunizable diseases	0-74	032, 033, 037, 045, 055, 056, 070.0, 070,1, 070.2-070.3, 072	A35, A36, A37, A49.2, A80, B05, B06, B15, B16, B17,0, B18,0-B18,1, B26	0,02	0,19

Data sorted descendent by age-standardized mortality rates in the United States.

**Table 3:** Classification of amenable causes of death according to Strategy 1 (literature review and empirical analysis), Strategy 2 (expert assessment), and the combined consensus.

Cause of death	Age	Strategy 1 Literature review and empirical analysis^*∗*^	Strategy 2 Expert assessment^*∗∗*^	Combined consensus for the two strategies Priority given to *high- efficacy *group in Strategy 1 and more than 85% in Strategy 2
Abdominal hernia	<75	G1 [7, 9, 29, 30]	100%	High efficacy

Appendix diseases	<75	G1 [7, 9, 29, 30]	100%	High efficacy

Maternal mortality	All	G1 [31–33]	100%	High efficacy

Perinatal mortality	-	G1/G2 [7, 9, 34–38]	100%	High efficacy

Cholecystitis/Cholelithiasis	<75	G1 [29, 30]	91%	High efficacy

Immunizable diseases	<75	G1 [29, 30]	91%	High efficacy

Asthma	5-49	G1 [7, 9, 12, 29, 30, 39, 40]	89%	High efficacy

Benign prostate hyperplasia	<75	G1 [29, 30]	89%	High efficacy

Peptic ulcers	<75	G1 [7, 9, 29, 30, 41–43]	89%	High efficacy

Pernicious anemia	<75	G1 [29, 30, 39]	89%	High efficacy

Thyroid diseases	<75	G1 [7, 9, 12, 29, 30, 39]	89%	High efficacy

Tuberculosis	<75	G2/G1 [44–50]	89%	Medium-low efficacy

Chronic rheumatic heart disease	<75	G2/G1 [7, 9, 29, 30, 51, 52]	67%	Medium-low efficacy

Diabetes mellitus	<50	G2/G1 [7, 9, 29, 30, 53–55]	67%	Medium-low efficacy

Hypertension	<75	G2/G1 [7, 9, 29, 30, 39, 56]	67%	Medium-low efficacy

Pneumonia, Acute Respiratory Infections and Influenza	<75	G2/G1 [29, 57–59]	67%	Medium-low efficacy

Congenital cardiovascular anomalies	<75	G2/G1 [29, 30, 51]	33%	Medium-low efficacy

Cervical cancer	15-74	G2/G1 [9, 60, 61]	22%	Medium-low efficacy

Skin cancer (melanoma and non-melanoma)	<75	G2 [29, 30, 60]	22%	Medium-low efficacy

Uterine cancer	15-74	G2 [7, 9, 29, 30]	22%	Medium-low efficacy

Cerebrovascular disease	<75	G2 [7, 9, 29, 30]	11%	Medium-lowefficacy

Testicular cancer	<75	G2/G1 [7, 9, 60, 62, 63]	11%	Medium-low efficacy

Hodgkin's disease	<75	G2/G1 [7, 9, 60, 64, 65]	0%	Medium-low efficacy

Leukemia	<15	G2 [7, 9, 60, 66]	0%	Medium-low efficacy

Ischemic heart disease	35-75	G2 [7, 9, 29, 30, 67]	0%	Medium-low efficacy

Breast cancer (women)	<75	G2 [7, 9, 60, 68]	0%	Medium-low efficacy

^*∗*^G1: amenable causes with medical interventions of *high efficacy*; G2: amenable causes with medical interventions of* medium-low efficacy*; G1/G2 or G2/G1: uncertain level of efficacy but priority given to the first. Three-way categories were based exclusively on the literature review.

^*∗∗*^Percentage of professionals who classified each amenable cause of death as having an intervention belonging to the highest level of efficacy.
